# Long-distance impact of Iceland plume on Norway’s rifted margin

**DOI:** 10.1038/s41598-017-07523-y

**Published:** 2017-09-04

**Authors:** Alexander Koptev, Sierd Cloetingh, Evgueni Burov, Thomas François, Taras Gerya

**Affiliations:** 1 Sorbonne Universités, UPMC Univ Paris 06, CNRS, Institut des Sciences de la Terre de Paris (iSTeP), 4 place Jussieu, 75005 Paris, France; 2 Department of Earth Sciences, Utrecht University, Utrecht, Netherlands; 3 ETH-Zurich, Institute of Geophysics, Sonnegstrasse 5, Zurich, Switzerland

## Abstract

Results of a 3D modeling study inspired by recent seismic tomography of the Northern Atlantic mantle suggest that a complex pattern of hot mantle distribution with long horizontal flows originating from the Iceland mantle plume has been the norm in the geological past. In the Northern Atlantic the Iceland plume has a strong long-distance impact on intraplate deformation affecting both onshore and offshore parts of Norway’s rifted margin. As a result, this margin is characterized by large magnitude differential topography sustained over at least several tens of Myr. Here we use high-resolution 3D thermo-mechanical modeling to demonstrate that the long-distance plume impact can be explained by its fast lateral propagation controlled by pre-existing lithosphere structures. Numerical models show that these structures strongly affect the style of horizontal flow of plume head material. This results in long-distance propagation of hot material emplaced at the lithosphere-asthenosphere boundary causing long-wavelength anomalies in onshore topography of Norway’s rifted margin. Short-wavelength offshore topographic domes are likely caused by joint occurrence of plume-related thermal perturbations and gravitational forces related to plate thickening (ridge push). Our 3D modeling brings together plume impingement, spreading ridge dynamics, and the formation of anomalous intraplate structures offshore Norway in one scenario.

## Introduction

Rifted continental margins form as a consequence of continental break-up. A number of factors, including the rigidity of the extending continent, the presence of thermal anomalies in the upper mantle, changes in plate-tectonic forces affecting stress levels and strain rates in the lithosphere are likely to have contributed to the observed spectrum in margin formation and evolution^1–3^. Many rifted margins are characterized by pronounced differential topography^4^.

A growing body of high quality new data is accumulating for the deep structure and vertical motions of rifted margins^1, 5^. These new data sets demonstrate pronounced deviations from classic rifting models, predicting steady post-rift subsidence patterns, controlled by thermal cooling during the post-rift phase. Post-rift erosion of sediments appears also to be more common than expected on the base of classic rifting models^4^. At the same time, a large body of new observations for in particular the Northern Atlantic margins and the margins of the Mediterranean^6^ provides ample evidence for post-rift compressional reactivation of rifted margins. Another striking observation in many rifted margin systems is the frequent close association with major thermal perturbations and associated volcanism at the transition of syn-rift and post-rift phase^1, 2^. The consequences of such perturbations, with characteristic time scales of several tens of Myr obviously are also manifest in the post-rift stage^1^.

**Figure 1:**
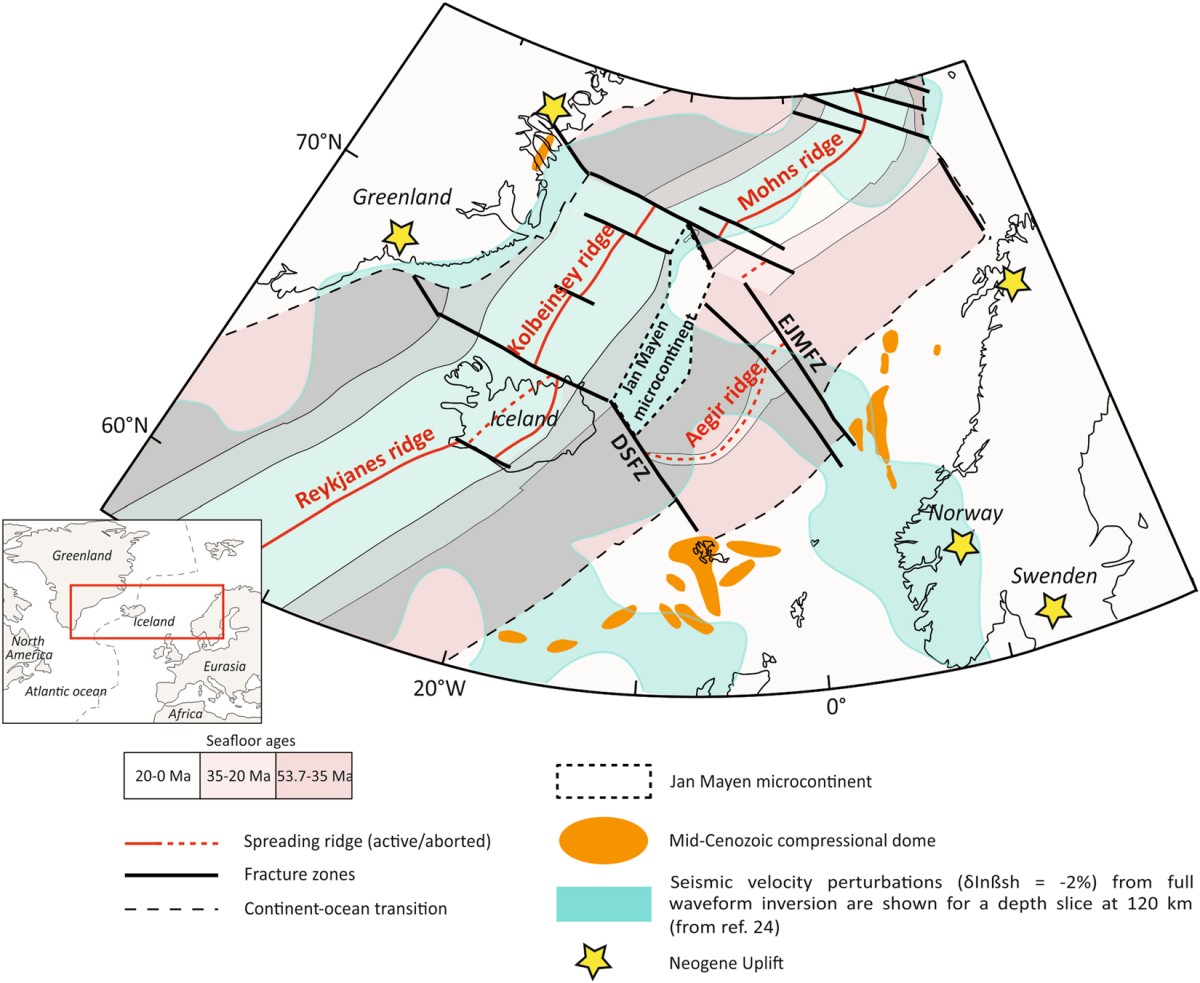
Tectonic setting of North Atlantic region (modified from ref. 22, Fig. 1) and low velocity anomaly at 120 km-depth^24^. *Abbreviations:* EJMFZ,

Plume-lithosphere interactions also have a profound impact on the dynamics and topographic expression of rifting^7–9^. It appears that plume emplacement affects not only the area overlying the initial position of the plume but impacts a much wider area. For example, Cenozoic magmatism in the African continent has been explained by impact of a single superplume^10^. An even more challenging example for testing models for plume-lithosphere interaction is the Northern Atlantic^11, 12^. This area has been affected by Late Cretaceous-Early Cenozoic continental break-up preceded by more than 200 Myr of rifting and is probably the best-documented volcanic rifted margin in the world^13, 14^. Striking lithospheric-scale contrasts occur between areas underlain by continental lithosphere including Iceland and surrounding areas^15^ and newly-created oceanic lithosphere, often subjected to jumps of spreading ridges and segmented by transform faults (Fig. 1). Enigmatic features in the Northern Atlantic margin system, especially in view of the overall success of classic rifting models to assess the first order features of rifted margin evolution, include the North Atlantic Large Igneous Province along both margins^16–18^, anomalous elevated topography^4, 19, 20^ and Mid-Cenozoic compressional domes offshore the Mid-Norway margin^21^ and the British Isles^22^.

A qualitative model has been proposed^23^ for asthenospheric diapirism as an explanation for Cenozoic post-rift domal uplift of North Atlantic margins. Since this early work significant advances have been made in quantitative studies of upper mantle and crustal structure of the Northern Atlantic as well as in new concepts of thermo-mechanical modelling including implementation in 3D fully-coupled lithosphere-scale models^7–9^.

Seismic tomography^24^ has demonstrated that the Iceland plume is not only extending along the mid-oceanic ridge but also in perpendicular directions, with sidelobes of the Iceland plume detected below the southern Scandes and British Isles (Fig. 1), whereas other studies have emphasized plume material moving northward towards the Greenland margin^25–27^.

Two models have been proposed to explain mantle-lithosphere interaction: a “bottom-up” model referring to a vertical upwelling of deep hot material^28^ probably anchored in the lowermost mantle^29^, and a “top-down” plate-driven model in which rifting of the lithosphere is controlled by shallow mantle processes^30^. The nature of plume-lithosphere interactions in the Northern Atlantic is not only bottom-up controlled by spatial and time-varying distributions of plume-related mantle thermal anomalies, but also in a top-down mode^1, 10^ by spatial variations in overlying lithospheric structure. The commonly made assumption of a uniform lithosphere at the site of future break-up^7, 31–34^ that could result in symmetrical radial patterns^35^ sometimes complicated by viscous fingering^12^ is not realistic in view of abundant evidence from the geological record that incipient rifts and rifted margins are usually localized at suture zones separating stronger lithosphere. Examples include the Caledonides suture, adjacent to cratonic lithosphere, localizing Devonian and Permo-Triassic rifting and subsequent continental break-up around 55 Ma in the Northern Atlantic^13, 36^ and the rift systems created at the edges of the African cratonic lithosphere^8, 9, 37, 38^.

So far these bottom-up^7^ and top-down^10^ controls on plume emplacement have been pursued separately. Our study is a first attempt to combine bottom-up and top-down approaches for volcanic rifted margin systems by fully-coupled 3D thermo-mechanical models of plume-lithosphere interactions^7–9, 39^.

Our main objective is to quantify the consequences of initial rifted margin lithospheric structure and configuration for onshore and offshore segments of the margin system affected by multidirectional mantle flow in the asthenosphere, propagating over hundreds kilometers from the area of initial plume emplacement. To this aim we first present the generic features of the model, followed by a comparison of our model findings with pertinent observations from the well-studied Northern Atlantic volcanic rifted margin province^13–18, 22, 36^.

To investigate the impact of the Iceland plume on post-breakup evolution of the Norwegian-Greenland Sea after its opening in the Early Eocene, the setup of the performed 3D models mimics roughly the relative position of continental and oceanic lithosphere in the Northern Atlantic region at 30–35 Ma. At this time the center of the Iceland plume has been aligned with the Reykjanes Ridge evolving during the Early Eocene (ref. 22; Supplementary Figure 1). The numerical model relies on two main assumptions: (1) that two main fracture zones (the East Jan Mayen Fracture Zone (EJMFZ) and the Denmark Strait Fracture Zone (DSFZ), as shown in Fig. 1) were active in Oligocene, and (2) the tomographic model^24^ images the main characteristics of the present day extent of the sublithospheric plume material. We realize that both assumptions have their shortcomings. For example, it is well known that the EJMFZ is long lived, and it can be observed in all remote sensing and geophysical data^40^. On the opposite, although a shorter eastern segment – the Iceland-Faroe Fracture Zone (IFFZ) –can be followed on present day oceanic crust, most of the DSFZ is less expressed in the present day bathymetry. Note, however, that despite the DSFZ cannot be mapped directly, it corresponds for most of its length to significant (several hundred km) horizontal offsets (Fig. 1).

**Figure 2:**
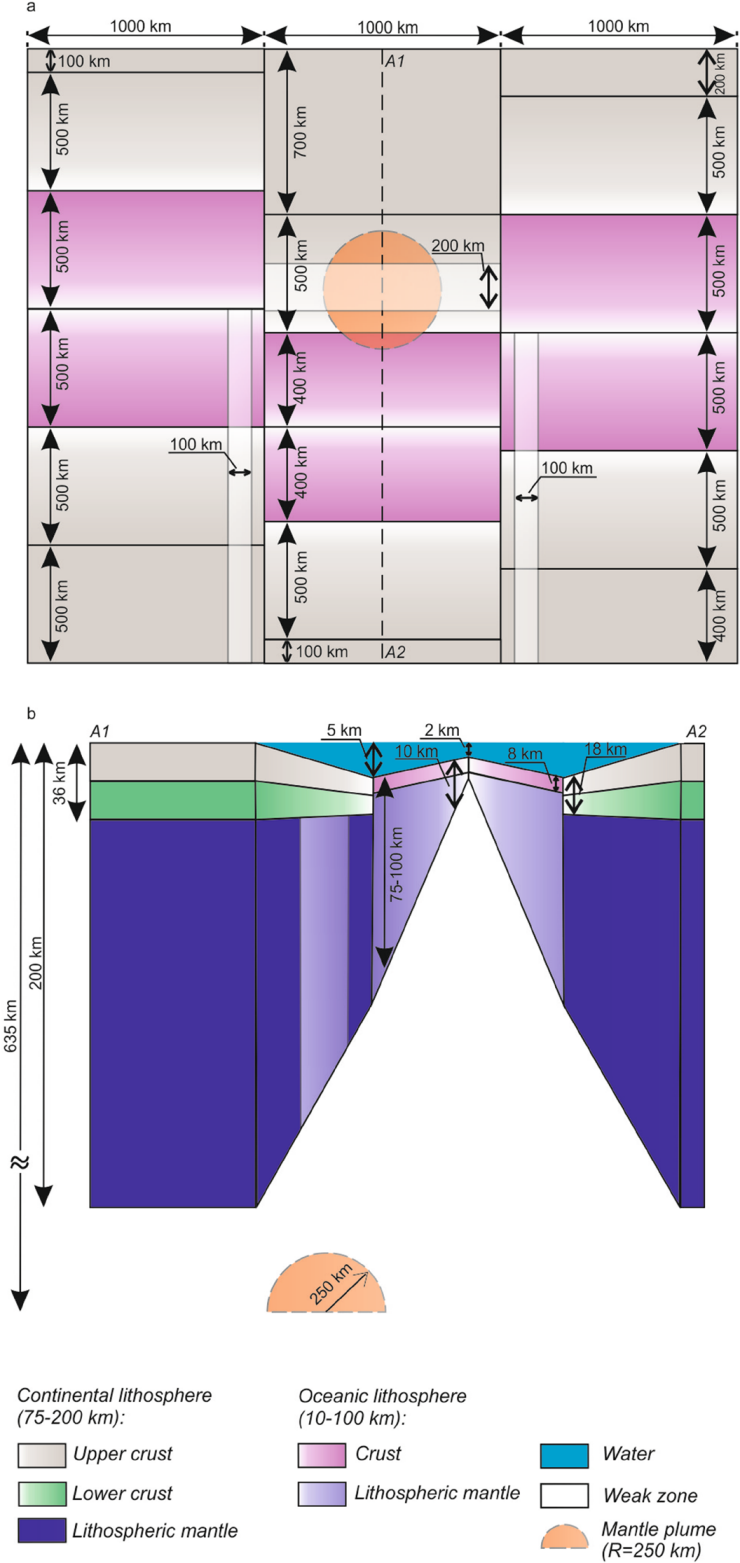
Initial 3D model setup corresponding to plate-tectonic reconstructions for the Northern Atlantic at 35 Ma (ref. 22; see Supplementary Figure 1): (**a**) plan-view: gradient colours from white to rose and grey illustrate gradual transition from minimum to maximum thickness values for continental and oceanic lithosphere, respectively; weak zones of lower lithospheric thicknesses are shown by transparent white; position of vertical cross-section A1–A2 is indicated by dashed line; (**b**) vertical cross section A1–A2: lithospheric thickness varies from 10 km to 75–100 km and from 75–100 km to 200 km for oceanic and continental lithosphere, respectively; oceanic crust is characterized by constant thickness (8 km) while continental crust is thickening from 18 to 36 km; a weak zone corresponding to lithosphere thinning (up to 90 km) within continental lithosphere is situated above a 250 km-radius mantle plume following evidence for a lithosphere weakness zone above the Iceland plume resulting in a plume-related jump of spreading axis from the Aegir ridge to the Kolbeinsey ridge at 35 Ma (ref. 22). Transform-parallel weak zones correspond to two main fracture zones in the Northern Atlantic: the East Jan Mayen Fracture Zone and the Denmark Strait Fracture Zone (Fig. 1). Lithospheric thinning of the lithosphere – corresponding to weak zones as well as their width – is implemented on the base of complementary 2D and 3D numerical tests (Supplementary Figure 4). See Methods for more details on model setup.

**Figure 3:**
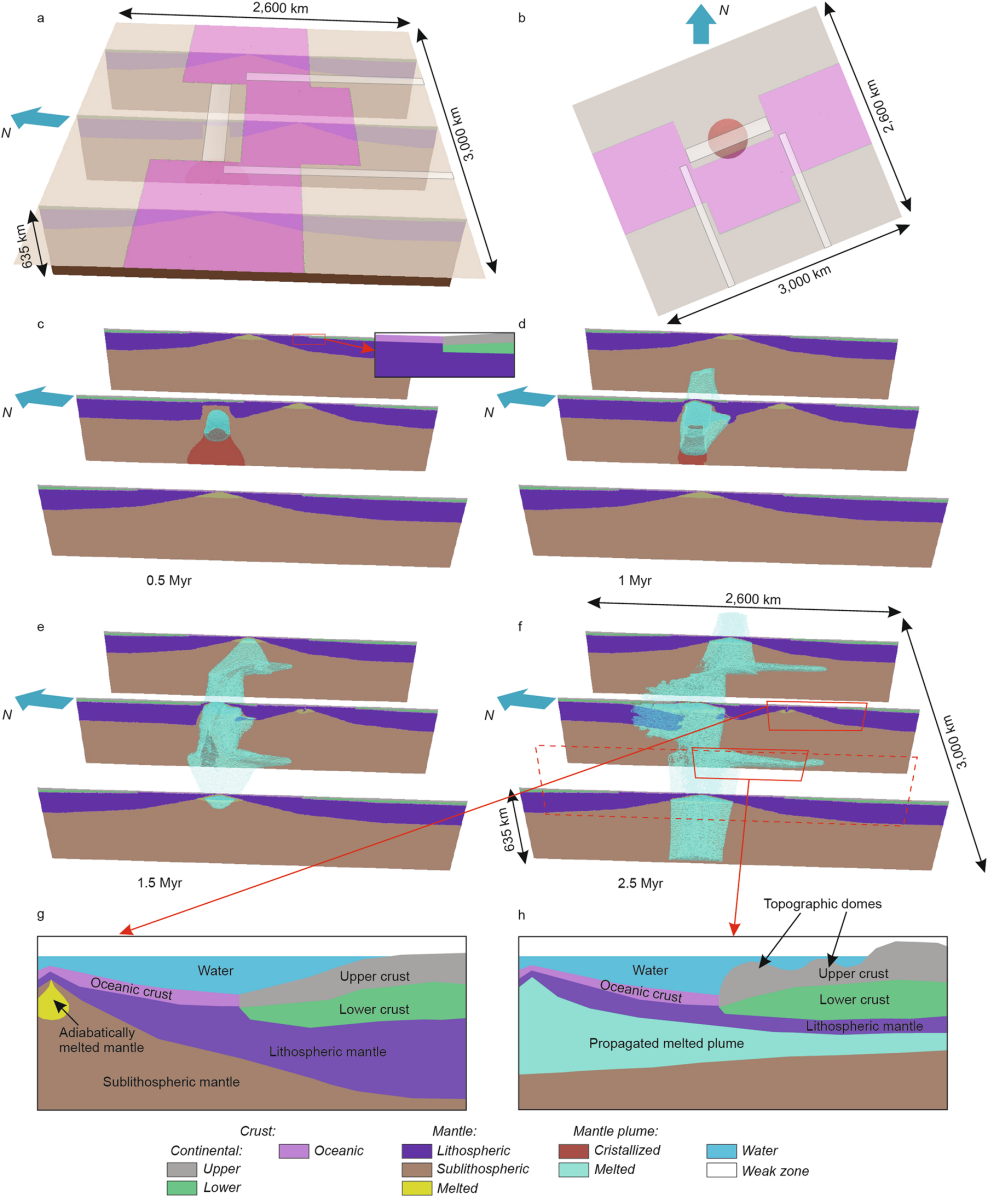
3D numerical experiment: (**a**,**b**) initial configuration: 3D projection (**a**) and plan-view (**b**); (**c**–**f**) 3D view of model evolution: ascent of the mantle plume up to the bottom of the lithosphere (**c**), symmetric lateral spreading of the plume material (see also Supplementary Figure 2) along pre-imposed NE-SW “weak” zone (**d**), onset of mantle plume propagation in two directions: NE-SW along mid-oceanic ridges and south-eastward along transform faults following another two zones of pre-imposed lithospheric thinning (**e**) and resulting distant (~1000 km) south-eastward propagation of the plume material (**f**); (**g**–**h**) two schematic sections for the southern part of the North Atlantic rifted margin province at locations without (**g**) and with (**h**) plume emplacement. The presence of mantle plume material underneath initially thinned lithosphere leads in this scenario to formation of significant differential offshore topography and elevated onshore margin topography (see Supplementary Figure 3) due to thermal weakening and ridge-push related compressional forcing. In contrast, the rifted margin at locations without mantle plume does not show considerable topography variations (Supplementary Figure 3) even despite larger ridge push forces due to greater contrast in lithosphere thickness. Zoom (top right inset of “c”) shows initial crustal structure at the rifted margin. Red dashed box refers to full-sized vertical cross-section through a SE-propagated lobe of molten mantle plume with its most representative part shown schematically below (**h**).

The model geometry is made up of three 1000 km-wide segments of oceanic lithosphere created by sea-floor spreading embedded into surrounding continents, whereas the central segment is shifted to the south-east with respect to the western and the eastern segments (Figs 2 and 3a,b). The mantle plume has been seeded by a thermal anomaly at the model box bottom underneath the transition zone between continental and oceanic lithosphere in the central segment. Following evidence for localized lithosphere-scale weaknesses above mantle plumes resulting in a plume-related jump of spreading axis from the Aegir ridge to the Kolbeinsey ridge, we have incorporated a 200 km-wide weak zone corresponding to thinner (90 km) lithosphere above the initial mantle plume. As shown by previous numerical models^41^, the thermal structure beneath oceanic transform faults is characterized by elevated temperatures associated to enhanced mantle upwelling that permits to sustain localized deformation within transforms for a long-time. Thus, another two 100 km-wide zones of local lithospheric thinning have been incorporated within the western and eastern segments of continental and oceanic lithosphere along transform faults south of the mantle plume corresponding to the East Jan Mayen Fracture Zone and the Denmark Strait Fracture Zone^14^. As shown in previous numerical studies^7^, interaction of an upwelling mantle plume with homogenous lithosphere leads to axisymmetric flow of plume head material emplaced at the lithosphere-asthenosphere boundary. Thus, incorporation of these pre-imposed weak zones appears to be a top-down prerequisite for localized flow of mantle plume material manifested by spatial patterns of low seismic velocities inferred from tomography^24^. The mechanical boundary conditions of the model are free slip at all boundaries that refers to absence of any pre-imposed specific kinematics.

After quick (~0.5 Myr) upwelling up to the lithospheric bottom (Fig. 3c), the mantle plume spreads laterally underneath continental lithosphere (Fig. 3d) following a pre-defined NE-SW “weak” zone in the central part of the model (Fig. 2). Once the plume has reached adjacent oceanic segments, it starts to propagate in two perpendicular directions: one part of plume material continues to spread in a NE-SW direction along mid-oceanic ridges, but another one flows to the south-east along transform faults (Fig. 3e) following two NW-SE zones of pre-imposed lithospheric thinning (Fig. 2).

**Figure 4:**
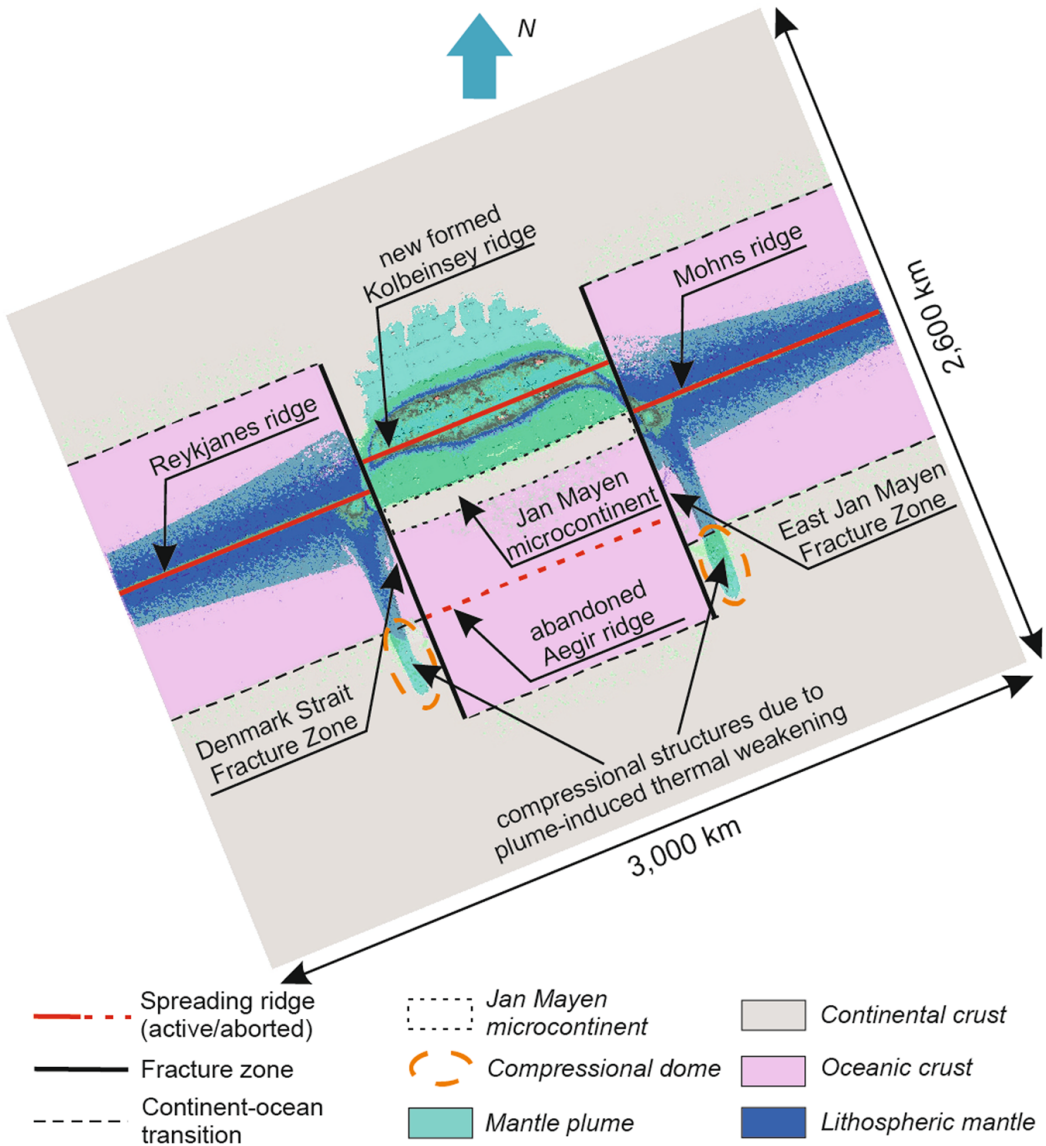
Plan-view of resulting components distribution. Horizontal slice at a depth level of 10 km shows distribution of continental and oceanic crust, lithospheric mantle and mantle plume material. Note remote south-eastward plume material propagation up to continental segments. This long-distance plume impact is a result of the top-down control of plume propagation by pre-existing lithosphere structures. Flow of plume material perpendicular to the spreading ridge along two weak transform faults, in combination with ridge push, is proposed to be responsible for the formation of intraplate topographic domes (see Supplementary Figure 3), as found offshore Norway and the British Isles (see Fig. 1).

In the central part of the model domain, plume-induced continental break-up (Fig. 3e) leads to formation of a new spreading axis following pre-imposed NE-SW “weak” zone and resulting in separation of a continental micro-block from the northern continent (Figs 3f and 4). This reproduces in detail the north-east propagation of the Kolbeinsey Ridge and separation of the Jan Mayen micro-continent from East Greenland at ~30 Ma (refs 22 and 36 and references therein). Note, however, that modelling of post-breakup spreading and rift jump processes (see e.g. refs 42 and 43) is beyond the scope of this paper. As shown by previous studies on basic fluid mechanics of plume-ridge interaction, seafloor spreading can restrict the along-axis flow of the plume material^44–48^. Despite possible limitations due to neglecting of seafloor spreading in our study, a strong contrast between extremely high velocity of plume propagation (that is in the order of 20–40 cm/year) and slow spreading rate of the Kolbeinsey Ridge (<2 cm/year since 9.5 Ma; see refs 49–51) makes this assumption reasonable. Open questions remain also on critical weakness of pre-imposed zones required to evolve into new spreading centres and on the nature of the time lag between ridge relocation around Jan Mayen. Our modelling provides a set of snapshots illustrating the first-order features of North-Atlantic plate tectonic regimes, and it is not targeting for detailed time evolution. It is also noteworthy that northward penetration of hot plume into continental lithosphere results in downward displacement of lithospheric mantle material of the northern continental plate corresponding to plume-induced initiation of continental subduction^39, 52^ (Fig. 3f). Note, however, that this prediction appears at first sight not be applicable for the North Atlantic region.

The resulting distribution of plume material shows its distant (~1000 km) south-eastward propagation along transform faults (Figs 3f and 4) up to southern continental segments. Our overall findings are thus largely in agreement with patterns inferred from seismic tomography data mapping present-day Iceland plume material not only along the mid-ocean ridge but also extending into southern Norway and the British Isles along two ridge-perpendicular zones^24^ (Fig. 1). Note, however, that the extent of the mantle plume material as depicted by tomography^24^ (Fig. 1), is not flowing along the DSFZ but slightly south of it, which points to a more complex distribution of the actual weak zones channeling plume material than adopted in the present modeling.

A distant propagation of the Iceland plume head in the Northern Atlantic can be just an example of a more general phenomenon related to fast flow in the shallow mantle. For example, extremely high velocities (>10 cm/year) for lateral migration of plume-related hot material have been also reported in the Arabia-Anatolia-Aegean system^53^.

In plan-view comparison of modelling results with the geometry reconstructed for time-slices corresponding to tipping points in the evolution of the Northern Atlantic shows a striking similarity in terms of key features so far not linked in a quantitative framework (Fig. 4). These include the appearance of a newly-formed spreading axis, the separation of a micro-continent, fast along-axis flow (resulting in giant V-shaped features observed along the Reykjanes Ridge)^54–57^ and south-eastern propagation of two lobes of partially molten mantle plume material underneath the adjacent volcanic rifted Norway margin and the southern Scandes. This lateral penetration of the plume head into the south-eastern segments of the continental lithosphere (Figs 2 and 3a,b) has reached 400 km in length and several tens km in width, corresponding to observed spatial dimensions of lithospheric thinning under the southern Scandes detected by deep seismic profiling^58^, mantle seismic tomography^24^ and topography anomalies^19^. Our findings support a causal link between upper mantle “asthenospheric” flow and domal uplift of the Northern Atlantic margin proposed earlier^23^, providing a self-consistent quantitative mechanism incorporating the mode of upper mantle-lithosphere interaction in a rifted margin setting.

It is explicitly not our aim to reproduce crustal deformation and topography in detail given the resolution of the 3D models (about 10 km per grid cell). Nevertheless, the overall patterns predicted by the model appear to be interesting in view of providing a first-order explanation for some current enigmatic observations. This includes presence of long-wavelength (hundreds of km) anomalies in onshore topography of Norway’s rifted margin and short-wavelength offshore topographic domes with characteristic wavelengths of several tens of km (Fig. 1). Combination of ridge-push related NW-SE oriented compressional forcing with lithospheric weakening due to plume-related thermal perturbations might well be able to explain these long- and short-wavelength intraplate deformation features (Supplementary Figure 3). Spatial variations of the orientations of these structures suggest a 3D nature of their causal controls including rotation of the main axes of compression. In this context, ridge push forces will contribute to a transition to a compressional stress regime, as will be the effect of rift shoulder topography, promoting compression in the adjacent offshore depocentre^59^. In addition, plate reorganizations encountered in the post-rift evolution associated to continuous opening of the Northern Atlantic with changes in spreading rates also can lead to a further increase in the level of the stress field in this region^22^. Compressional structures offshore^6, 21^ have been widely documented with their wavelengths of the order of several tens of km, typical for crustal scale folding. As pointed out by several authors^59, 60^, stresses induced by topography build-up along margins can lead to strong feed-backs with plate motion changes. Lateral variations in mechanical structure across rifted margins as well as along strike segmentation inherited from the pre-rift phase probably play an important role, not only in their formation but also in their subsequent deformation history.

Both plume activity and resulting compressional reactivation can result in short-term and long-term deviations in vertical motions from predictions of classical rifting models. The initiation of differential topography and accelerations in subsidence and uplift can be of very short-term nature, with typical time scales in the order of a few Myr.

Modeling results presented here and their possible implication in the context of the Northern Atlantic rifted margin system are probably more than of only regional significance. Onshore deformation and uplift have been reported in many areas affected by continental rifting and it appears that outward flow of hot and shallow mantle after plume emplacement may propagate at high velocities in other parts of the globe such as the Afar-Arabia region^53^.

## Methods

### General overview

Investigating plume-lithosphere interactions requires a model that incorporates a thermo-rheologically realistic lithosphere fully coupled to mantle dynamics in three dimensions, encompasses a wide region, and includes the entire upper mantle. This infers large-scale 3D models with high tectonic-scale spatial resolution (of at least 10 × 10 × 10 km). The corresponding mesh dimensions are very important, on the order of 5 × 10^6^ elements, which implies unprecedented numerical efforts. We meet this challenge using and optimizing the staggered grid/particle-in cell viscous-plastic 3D code I3DELVIS^61^.

### Governing equations

The momentum, continuity and energy equations are solved by the numerical code I3ELVIS^61^. Its numerical schema is based on finite-differences with a marker-in-cell technique where physical properties are transported by Lagrangian markers that move according to the velocities field interpolated from the fixed fully staggered Eulerian grid^61, 62^.

The momentum equations are solved in the form of Stokes flow approximation:

1 $$\begin{array}{l}\frac{\partial {\sigma }_{xx}^{^{\prime} }}{\partial x}+\frac{\partial {\sigma }_{xy}^{^{\prime} }}{\partial y}+\frac{\partial {\sigma }_{xz}^{^{\prime} }}{\partial z}=\frac{\partial P}{\partial x},\\ \frac{\partial {\sigma }_{yx}^{^{\prime} }}{\partial x}+\frac{\partial {\sigma }_{yy}^{^{\prime} }}{\partial y}+\frac{\partial {\sigma }_{yz}^{^{\prime} }}{\partial z}=\frac{\partial P}{\partial y}-g\rho ,\\ \frac{\partial {\sigma }_{zx}^{^{\prime} }}{\partial x}+\frac{\partial {\sigma }_{zy}^{^{\prime} }}{\partial y}+\frac{\partial {\sigma }_{zz}^{^{\prime} }}{\partial z}=\frac{\partial P}{\partial z}\end{array}$$

where ${\sigma }_{ij}^{^{\prime} }$ are the components of the viscous deviatoric stress tensor, *P* is the dynamic pressure, *ρ* is the density and *g* is the acceleration due to gravity.

Conservation of mass is approximated by the continuity equation:

2 $$\frac{\partial {V}_{x}}{\partial x}+\frac{\partial {V}_{y}}{\partial y}+\frac{\partial {V}_{z}}{\partial z}=0,$$

where *V*_*x*_, *V*_*y*_ and *V*_*z*_ indicate the components of velocity vector.

The components of the deviatoric stress tensor are calculated using the viscous constitutive relationship between stress and strain rate for a compressible fluid^62^:

3 $${\sigma }_{ij}^{^{\prime} }=2\eta {\dot{\varepsilon }}_{ij},$$

where the components of shear strain rate tensor are:

4 $${\dot{\varepsilon }}_{ij}=1/2(\frac{\partial {V}_{i}}{\partial {x}_{j}}+\frac{\partial {V}_{j}}{\partial {x}_{i}}).$$

The model uses non-Newtonian visco-plastic rheologies where the viscosity for dislocation creep is defined as follow^63, 64^:

5 $$\eta =1/2{({A}_{D}exp(\frac{E+PV}{RT}))}^{\frac{1}{n}}{{\dot{\varepsilon }}_{II}}^{\frac{1-n}{n}},$$

where *T* is temperature, ${\dot{\varepsilon }}_{II}=\sqrt{1/2{\dot{\varepsilon }}_{ij}{\dot{\varepsilon }}_{ij}}$ is the second invariant of the strain rate tensor and *A*_*D*_, *E*, *V*, *n* and *R* are the material constant, the activation energy, the activation volume, the stress exponent and the gas constant respectively. The power-law rheology is key in the strong channeling behavior of the mantle plume.

Plasticity is implemented using the Drucker-Prager yield criterion^64^:

6 $${\sigma }_{yield}=C+Psin(\phi ),$$

where *C* and *φ* the residual rock strength and the internal frictional angle respectively that depend on the total plastic strain^65, 66^.

The mechanical equations are coupled with heat conservation equations:

7 $$\begin{array}{l}\rho {C}_{p}(\frac{\partial T}{\partial t})=-\frac{\partial {q}_{x}}{\partial x}-\frac{\partial {q}_{y}}{\partial y}-\frac{\partial {q}_{z}}{\partial z}+{H}_{r}+{H}_{a}+{H}_{s}\\ {q}_{x}=-k\frac{\partial T}{\partial x}\\ {q}_{y}=-k\frac{\partial T}{\partial y}\\ {q}_{z}=-k\frac{\partial T}{\partial z}\end{array}$$

where *C*_*p*_ is the heat capacity; *k* is the thermal conductivity, *H*_*r*_ is the radiogenic heat production and *H*_*a*_ and *H*_*s*_ are the contributions due to isothermal (de)compression (i.e., adiabatic heating/cooling) and the shear heating, respectively.

The code is fully thermo-dynamically coupled and accounts for mineralogical phase changes by thermodynamic solution for density, $\rho$ = *f* (*P*, *T*) obtained from optimization of Gibbs free energy for a typical mineralogical composition of the mantle, plume and lithosphere material^67^. Partial melting is taken into account using the most common parameterization^68, 69^ of hydrous mantle melting processes. For crustal rocks we use a simple Boussinesq approximation since phase transformations in these rocks are of minor importance for the geodynamic settings explored here.

The effectively free surface topography is reproduced by emplacement at the top of upper crust a 30 km-thick low-viscosity “sticky air” layer^70, 71^.

More detailed information on rheological and material properties can be found in our previous studies^7–9^.

### Model setup

3D model box is characterized by horizontal dimensions of 3000 × 2600 × 635 km and consists of 297 × 257 × 67 nodes offering spatial resolution of ca. 10 × 10 × 10 km per grid cell.

The initial setup consists of three 1000 km-wide zones, each of which contains oceanic lithosphere embedded into surrounding continents. Position of oceanic lithosphere within different zones refers to northern shift of western (500 km) and eastern (400 km) oceanic segments with respect to central one (Fig. 2a). Initial thickness of the oceanic lithosphere varies from 10 km underneath of the mid-ocean ridge up to 75–100 km near continental margin. Continental lithosphere has thickened from 75–100 km to 200 km within 500 km-long area adjacent to oceanic segments. Mantle plume has been seeded 200 km to north of the northern boundary of the central oceanic segment by a spherical (radius of 250 km) thermal anomaly of 370 °C temperature excess at the base of the model domain. In doing so, we have adopted the simplest symmetrical scenario. A stratified three-layer continental lithosphere is composed of an upper and lower crust and lithospheric mantle. Total thickness of bi-layer continental crust changes from 18 km to 36 km synchronously with lithospheric thickness variations (Fig. 2b). Felsic (wet quartzite flow law) and mafic (plagioclase flow law) rheology has been employed for upper and lower crust, respectively. Monolayer oceanic crust is characterized by mafic composition and constant thickness of 8 km. The ductile rheology of both continental and oceanic lithospheric mantle is controlled by dry olivine dislocation (dry olivine flow law), while sublithospheric mantle deforms predominantly by diffusion creep (dry olivine flow law as well). The initial thermal structure refers to a linear vertical gradient with 0 °C at the surface, 700 °C at the continental Moho, and 1630 °C at the model box bottom. The bottom of the continental and oceanic lithosphere corresponds to an initial isotherm of 1300 °C. We apply insulating (zero conductive heat flux) for all vertical boundaries. Free slip has been adopted as common boundary condition for all boundary elements. Free slip condition requires that the two non-orthogonal components of velocity do not change across the boundary whereas the normal one is zero^61^.

We have assumed a 200 km-wide weak zone corresponding to thinner (90 km) lithosphere above the initial mantle plume consistent with evidence for a lithosphere weakness zone above the Iceland plume resulting in a plume-related jump of spreading axis from the Aegir ridge to the Kolbeinsey ridge at 35 Ma (ref. 22). Another two 100 km-wide zones of local lithospheric thinning have been incorporated within western and eastern segments of continental and oceanic lithosphere along transform faults to the south of the mantle plume, that correspond to two main fracture zones within the studied area: the East Jan Mayen Fracture Zone and the Denmark Strait Fracture Zone (Fig. 1).

Prescribing of “weak seeds” by thermal perturbation associated to localized lithosphere necking is a standard approach for both analogue and numerical models of rifting and spreading^66, 72–78^.

Geometrical parameters of these weak zones including their width have been defined by detailed 2D and 3D parametrical analysis (see Supplementary Figure 4).

## Electronic supplementary material

Supplementary Information
