# Supplementary material for: Long-distance impact of Iceland plume on Norway’s rifted margin
Source: Sci Rep. 2017 Sep 4;7:10408. doi: 10.1038/s41598-017-07523-y (PMC5583333; doi:10.1038/s41598-017-07523-y)
Supplement: Supplementary file 1 — Supplementary Information [file 41598_2017_7523_MOESM1_ESM.pdf]

Long-distance impact of Iceland plume on Norway's rifted margin

Alexander Koptev[1], Sierd Cloetingh[2], Evgueni Burov[1†], Thomas François[2], Taras Gerya[3]

[1]Sorbonne Universités, UPMC Univ Paris 06, CNRS, Institut des Sciences de la Terre de Paris (iSTeP), 4 place Jussieu 75005 Paris, France

[2]Department of Earth Sciences, Utrecht University, Netherlands

[3]ETH-Zurich, Institute of Geophysics, Sonnegstrasse 5, Zurich, Switzerland

[†] Deceased 9 October 2015

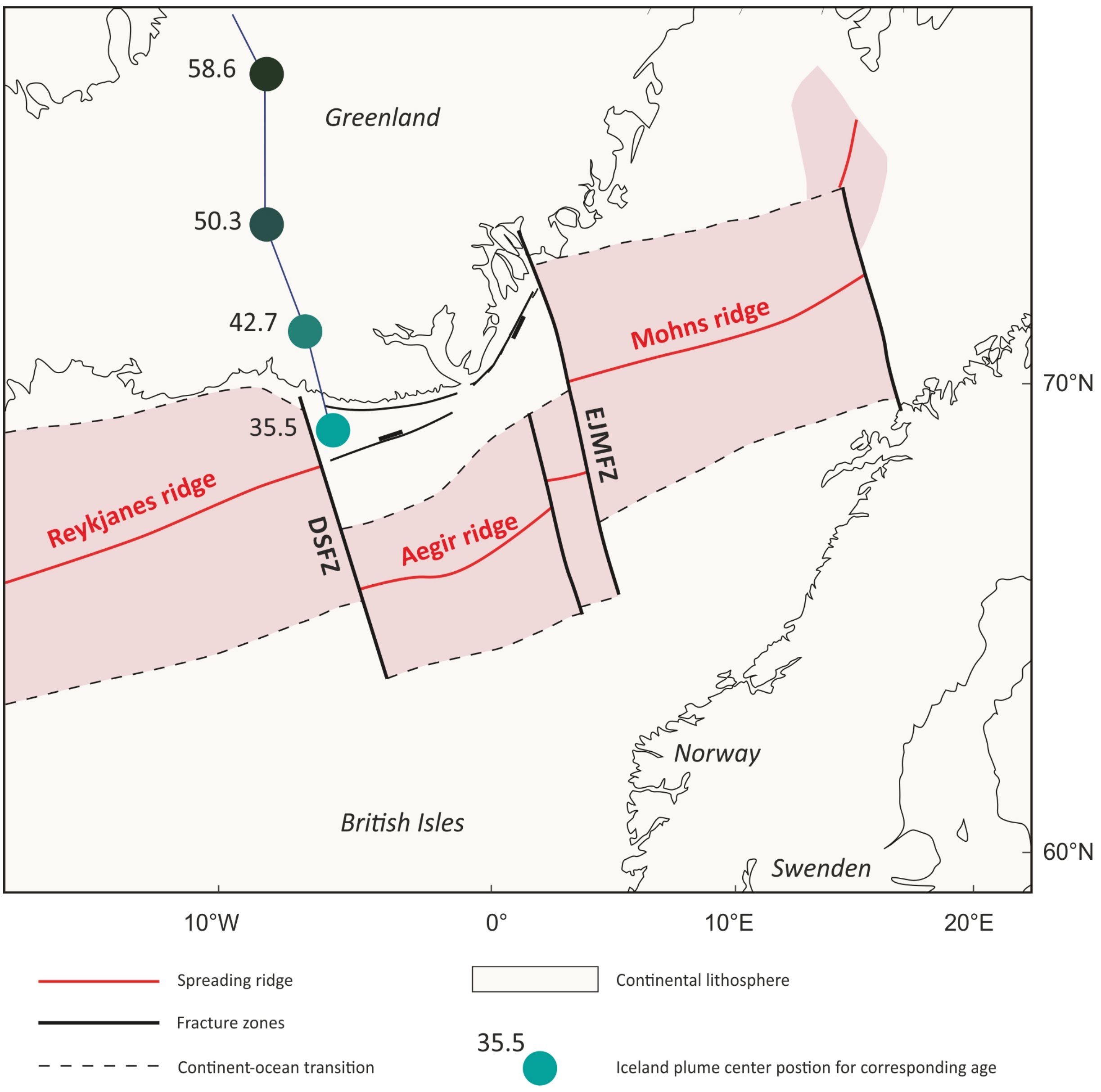

Supplementary Figure 1. Schematic plate-tectonic reconstruction of the Northern Atlantic, 35 Ma (modified from ref. 22, Figure 3b).

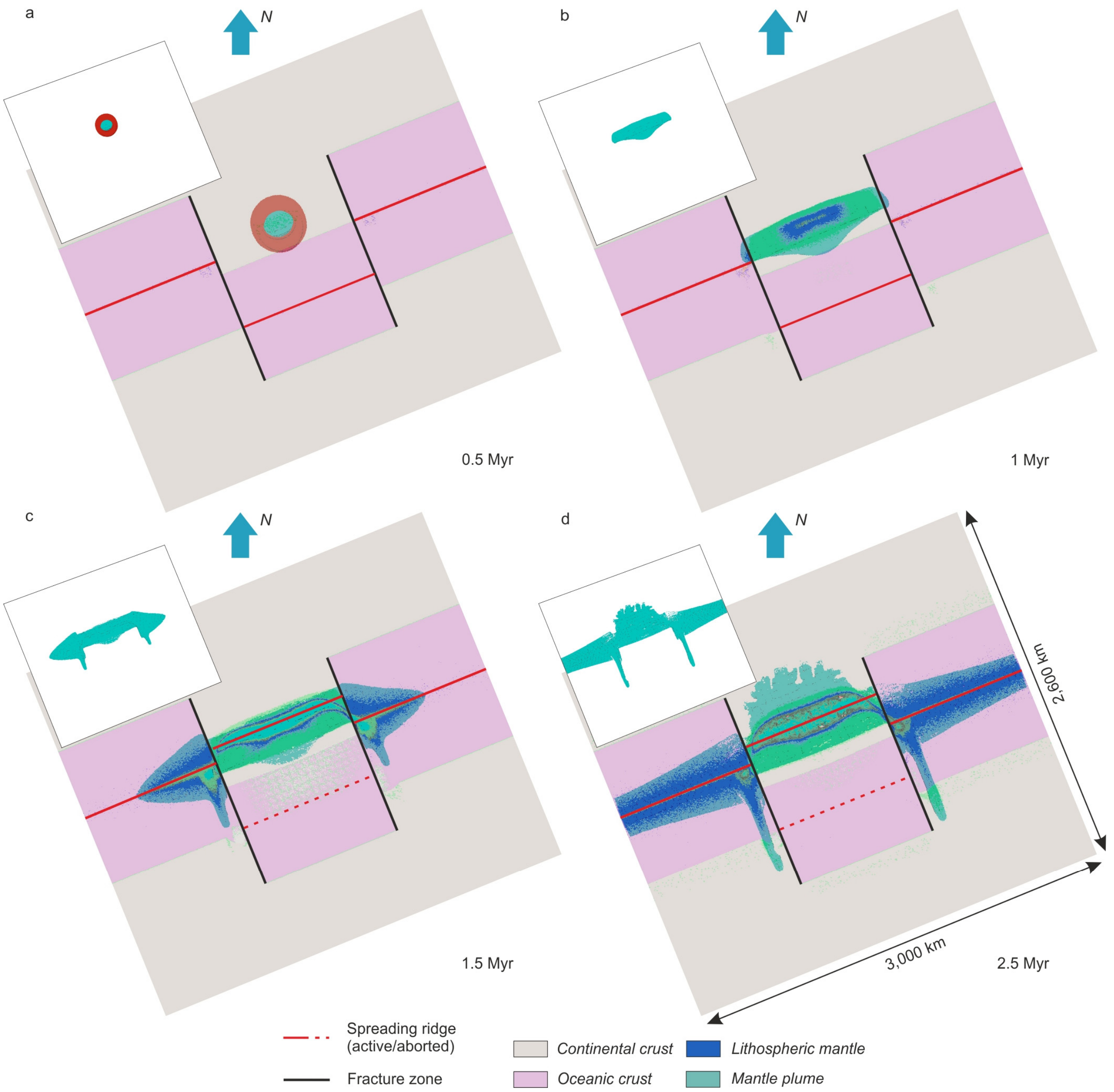

Supplementary Figure 2. Plan-view of 3D model evolution. Time slices shown here correspond to the ones displayed in Figure 3 (see Figure 3c-f). Note that "blue" material of the lithospheric mantle appears in bands along the ridge segments growing in length and width with time. This reflects local topography uplifts (see Supplementary Figure 3), raising the oceanic crust out of the horizontal slice which is replaced by lithospheric mantle or plume material flowing from beneath. Top left insets show the bulk of the plume material.

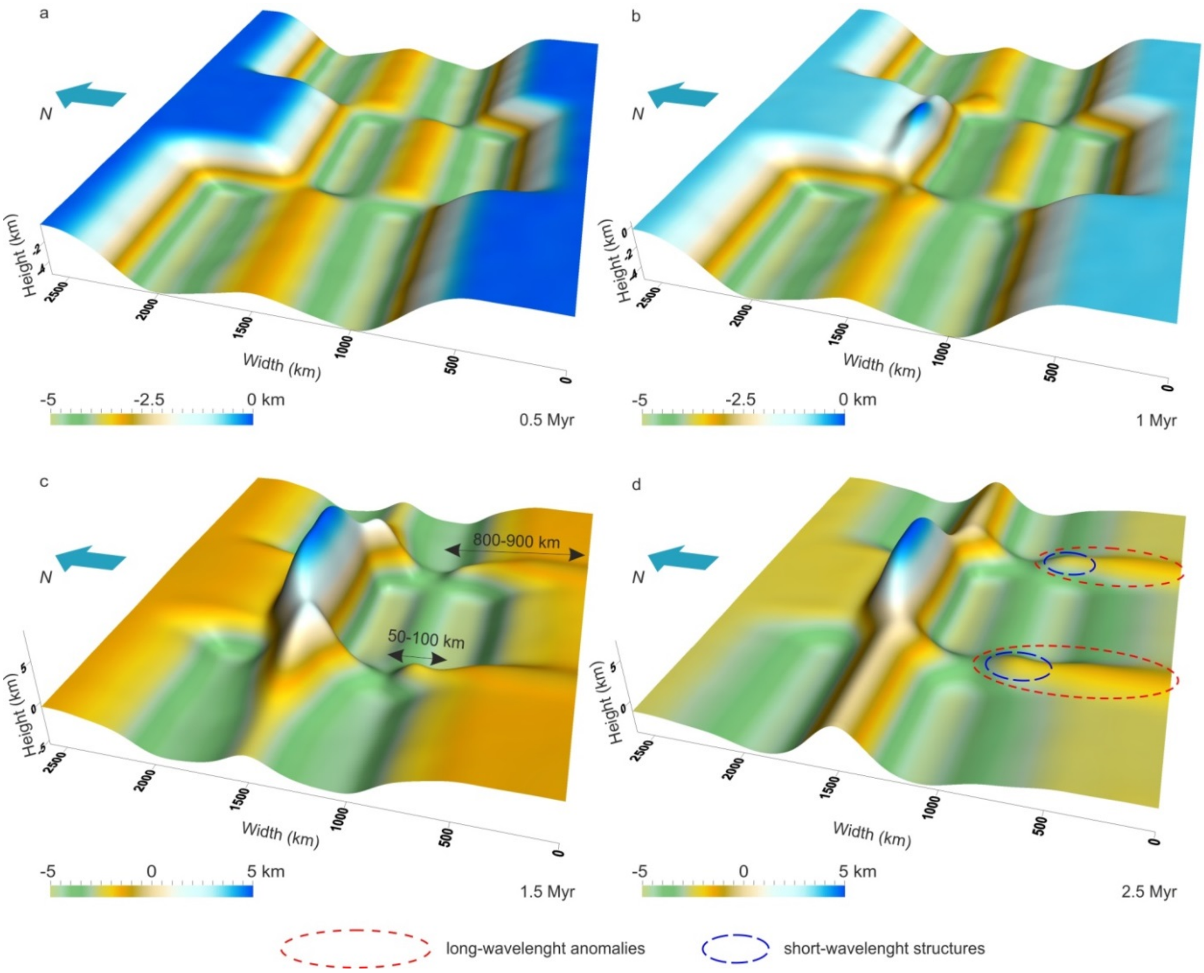

Supplementary Figure 3. Evolution of the modeled topography. The time slices shown here correspond to the ones displayed in Figure 3c-f and in Supplementary Figure 2. Note long-wavelength (hundreds of km) and short-wavelength (tens of km) topography anomalies within the southern segments of the continental lithosphere. Short-wavelength intraplate deformation might be explained by a combination of ridge-push forces and lithospheric weakening due to plume-related thermal perturbations of the lithosphere which are associated with a long-distance propagation of hot plume material in a SE direction (see Figure 3c-f, Figure 4 and Supplementary Figure 2). Significant surface uplift (up to 5 km in area of initial plume impact) results from buoyancy and dynamic push of mantle plume. Values of modeled topography are overestimated due to fixed (in normal direction) boundaries and neglect of erosional processes in the experiments. Note that more quantitative tests are needed to further explore the effects of plumes on topography for a detailed comparison with observations of rifted margin differential topography.

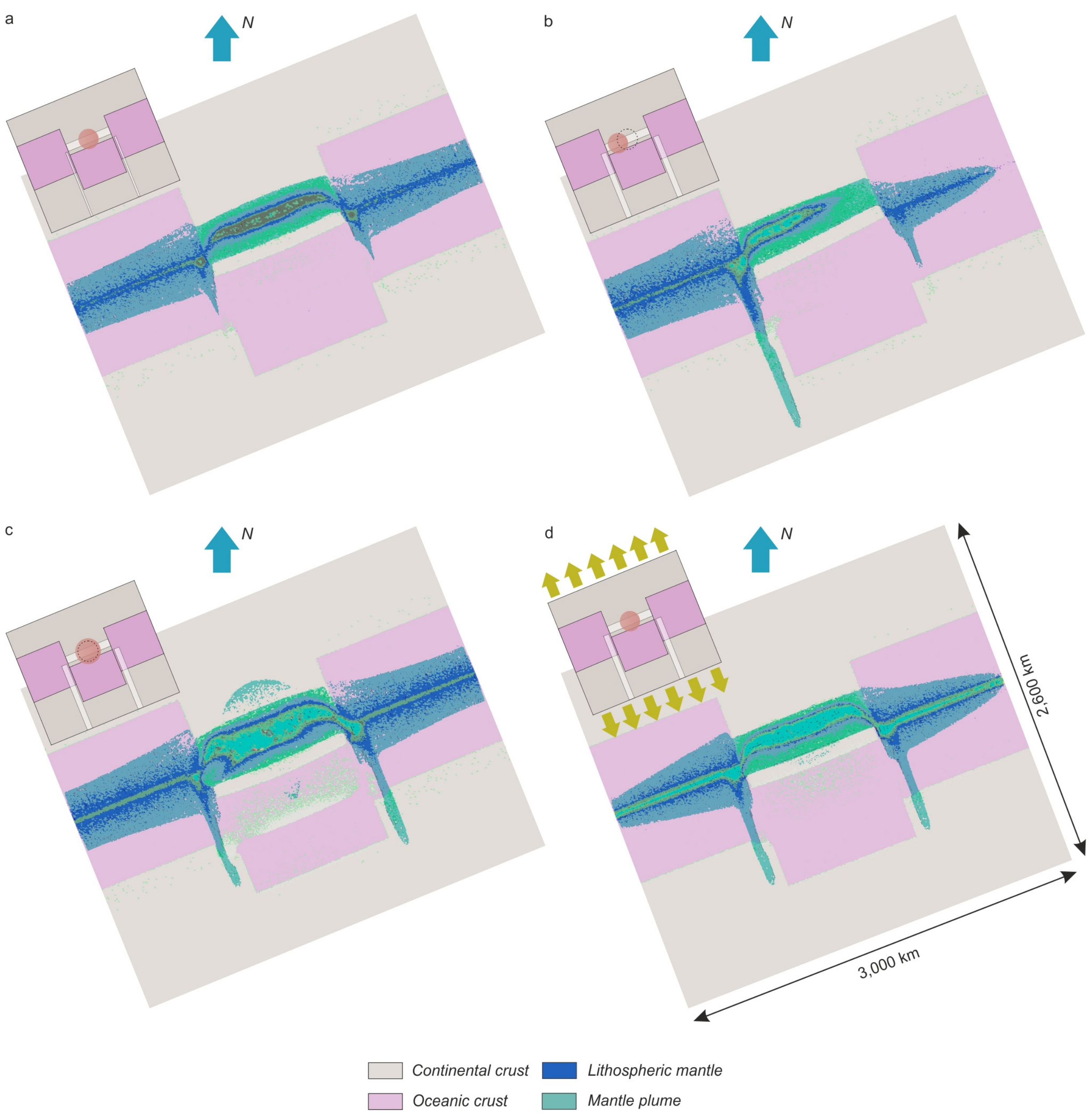

Supplementary Figure 4. Plan-view of resulting component distribution for the supplementary experiments: a) model with narrow (25-50 km-width instead of 100 km used in another experiments) weak zones that are pre-imposed along transform faults; this experiment does not show a distant south-eastward propagation of the plume material; b) lateral SW shift of initial plume position leading to distinct asymmetry in the final shape of the separated plume head; c) model with bigger mantle plume (radius of 300 km) resulting in final component distribution similar to that of the model with "reference" radius (250 km) of the plume anomaly (see Figures 3-4); d) model with imposed spreading velocities (half rate of 2 cm/year) showing long-distance bi-directional lateral migration of plume head material as in the case of the "reference" experiment without pre-defined far-field extension (Figures 3-4). It is noteworthy that 1) initial thickening of lithosphere within predefined weak zones up to 110-

120 km (instead of 90 km as adopted in experiments presented here) leads to restricted plume flow in a SE direction similarly to model with narrow weak zones (shown in “a”); 2) initial plume position appears to be a much more important controlling parameter than plume size or applied far-field extension (compare “b”, “c” and “d” with a “reference” case presented in Figures 3-4). Top left insets show schematically initial setup for each model (see Figure 3 for legend); “reference” size and position of the mantle plume is indicated by dashed circle.
